# Comparative root transcriptome of wild *Arachis* reveals NBS-LRR genes related to nematode resistance

**DOI:** 10.1186/s12870-018-1373-7

**Published:** 2018-08-06

**Authors:** Ana Paula Zotta Mota, Bruna Vidigal, Etienne G. J. Danchin, Roberto Coiti Togawa, Soraya C. M. Leal-Bertioli, David John Bertioli, Ana Claudia Guerra Araujo, Ana Cristina Miranda Brasileiro, Patricia Messenberg Guimaraes

**Affiliations:** 1 EMBRAPA Genetic Resources and Biotechnology, Brasilia, DF Brazil; 2 Universidade Federal do Rio Grande do Sul, Campus do Vale, Porto Alegre, RS Brazil; 3 INRA, Université Côte d’Azur, CNRS, ISA, Paris, France; 4 Center for Applied Genetic Technologies, University of Georgia, Athens, Georgia USA

**Keywords:** *Meloidogyne*, resistance genes, peanut, Root Knot Nematode, transcriptome

## Abstract

**Background:**

The Root-Knot Nematode (RKN), *Meloidogyne arenaria*, significantly reduces peanut grain quality and yield worldwide. Whilst the cultivated species has low levels of resistance to RKN and other pests and diseases, peanut wild relatives (*Arachis* spp.) show rich genetic diversity and harbor high levels of resistance to many pathogens and environmental constraints. Comparative transcriptome analysis can be applied to identify candidate resistance genes.

**Results:**

Transcriptome analysis during the early stages of RKN infection of two peanut wild relatives, the highly RKN resistant *Arachis stenosperma* and the moderately susceptible *A. duranensis*, revealed genes related to plant immunity with contrasting expression profiles. These included genes involved in hormone signaling and secondary metabolites production and also members of the NBS-LRR class of plant disease resistance (R) genes. From 345 NBS-LRRs identified in *A.duranensis* reference genome, 52 were differentially expressed between inoculated and control samples, with the majority occurring in physical clusters unevenly distributed on eight chromosomes with preferential tandem duplication. The majority of these NBS-LRR genes showed contrasting expression behaviour between *A. duranensis* and *A. stenosperma*, particularly at 6 days after nematode inoculation, coinciding with the onset of the Hypersensitive Response in the resistant species. The physical clustering of some of these NBS-LRR genes correlated with their expression patterns in the contrasting genotypes. Four NBS-LRR genes exclusively expressed in *A. stenosperma* are located within clusters on chromosome Aradu. A09, which harbors a QTL for RKN resistance, suggesting a functional role for their physical arrangement and their potential involvement in this defense response.

**Conclusion:**

The identification of functional novel R genes in wild *Arachis* species responsible for triggering effective defense cascades can contribute to the crop genetic improvement and enhance peanut resilience to RKN.

**Electronic supplementary material:**

The online version of this article (10.1186/s12870-018-1373-7) contains supplementary material, which is available to authorized users.

## Background

Root-Knot Nematode (RKN), *Meloidogyne arenaria*, affects peanut production in the US, Africa and Asia and can result in significant yield losses [1]. Whilst the cultivated species (*Arachis hypogaea*) has low levels of RKN resistance, peanut wild relatives (*Arachis* spp.) show rich genetic diversity harboring high levels of resistance to many pathogens and environmental constraints [2–5]. All *A. hypogaea* cultivars with improved RKN resistance were developed through the introgression of two segments of a single chromosome from the wild relative *A. cardenasii* [6–9], thus making the identification of additional resistance sources critical to avoid resistance breakdown and assure breeding advances.

The wild species *A. stenosperma* harbors high levels of resistance against the peanut RKN *M. arenaria* and various foliar fungi [10–14]. Overall, the penetration and development of the RKN in the resistant species was reduced in comparison to the susceptible, with dark blue cytoplasm and altered organelle structures observed in the central cylinder, indicating a hypersensitive-like response (HR). In the moderately susceptible *A. duranensis*, the nematode reproduction occurs, albeit at lower levels and with a development delay when compared to the susceptible *A. hypogaea* [12].

In response to RKN infection, our previous studies showed that *A. stenosperma* bares a mechanism of resistance known as the Hypersensitive Response (HR) [15, 16], which is often triggered by Resistance genes (R) [17]. In addition, the recent identification of four QTLs in *A. stenosperma* reducing RKN galling and egg production [12], reinforces the importance of this species as a new source of resistance.

Plant R genes are key to many plant-pathogen interactions, as they enable plants to recognize pathogens and activate inducible defenses which often culminate in rapid HR response [18]. The vast majority of plant R genes are NBS-LRR, as they encode proteins with an amino-terminal variable domain, a central Nucleotide Binding Site (NBS) and a carboxy-terminal Leucine Rich Repeats (LRR) domain [19]. Both classes of NBS-LRR genes (TIR-type and CC-type/non-TIR) are commonly present in multigene clusters in plant genomes and can occur as true alleles across naturally variant genetic backgrounds [17]. Although many plant genomes have been sequenced and thousands of putative R genes, the Resistance Gene Analogs (RGAs) identified, only a relatively small number of R genes associated with nematodes resistance have been isolated and fully characterized [20, 21].

In *Arachis*, the first survey of RGAs using degenerate primers targeting the NBS domain revealed 78 NBS-LRR encoding sequences with unknown function [22]. Later, hundreds of RGAs were isolated from different peanut cultivars using the same strategy and genome BAC sequencing [23–25]. More recently, a genome-wide analysis of NBS-LRR genes in the peanut progenitor wild species, *A. duranensis* and *A. ipaënsis*, identified over 300 representatives classified in four NBS-LRR family types [26, 27]. However, only a relatively small number of NBS-LRR involved in the responses to pathogens has been unveiled [27, 28]. The analysis of *A. stenosperma* transcriptome identified several candidate genes involved in the defense signaling and response at the early stages of its incompatible interaction with RKN, including NBS-LRR genes [16], but the lack of a reference genome hampered the accurate identification and characterization of members of NBS-LRR and other complex gene families. The recent availability of the sequenced genomes of *A. duranensis* and *A. ipaënsis* [26], has greatly facilitated genome-wide studies in the genus [27, 29–31]. Nonetheless, no studies have yet contemplated the set of NBS-LRR genes expressed upon nematode infection.

The comparative transcriptome analysis of resistant and susceptible genotypes to different stresses has provided new insights into plant response mechanisms and can identify new sets of candidate resistance genes [32–38]. This approach has not yet been explored in wild *Arachis* species, but can be particularly fruitful, as these species have relatively limited transcriptome data available, still lack publicly available microarrays, reference transcriptomes and comprehensive transcripts datasets.

In this study, we investigated the expression profiles of candidate resistance genes to *M. arenaria* in two wild *Arachis* species with contrasting responses, the highly resistant *A. stenosperma* and the moderately susceptible *A. duranensis*, with the focus on the NBS-LRR class of R genes. Considering the narrow genetic base of peanut and the single RKN resistance source available in the crop cultivars, the identification and characterization of new resistance genes in wild genotypes will substantially contribute to expand the repertoire of resistances and secure their durability.

## Methods

### Plant material and Illumina sequencing

*Arachis duranensis* (accession K7988) roots challenged with *M. arenaria* race 1 were obtained as previously described [14], using four-week-old plants inoculated with 20,000 *M. arenaria* juveniles (J2). RNA was extracted from whole roots collected at 3, 6, and 9 days after inoculation (DAI) and non-inoculated plants as a control (Ctrl), using a modified lithium chloride protocol [39], and purified with Invisorb Plant RNA Mini Kit (Invitek, Berlin, Germany). Two independent biological replicates were produced by pooling equal amounts of total RNA per collecting point and cDNA was produced using the Super Script II enzyme and oligo (dT) 20 primer (Invitrogen, Carlsbad, CA, USA), according to manufacturer’s instructions. Eight paired-end libraries were constructed corresponding to the control and each of the three time points of the interaction: *A. duranensis* (DCtrl), 3DAI (DN3), 6DAI (DN6) and 9DAI (DN9) in biological duplicates. Libraries were sequenced in Hi-Seq 2000 at FASTERIS (www.fasteris.com), employing the mRNA-Seq and TruSeq (TM) SBS v5 protocols (Illumina, San Diego, CA). For *A. stenosperma* (accession V10309), we used Illumina transcript reads produced earlier using the same above conditions [16].

### Gene expression analysis

Illumina raw reads from *A. duranensis* and *A. stenosperma* were trimmed by Trimmomatic version 0.33 [40] and their quality checked by FastQC (http://www.bioinformatics.babraham.ac.uk/projects/fastqc). Cleaned high quality reads were mapped to the annotated reference genome of *A. duranensis* (accession V14167) [26] (https://peanutbase.org) using the default settings of GMAP/GSNAP package [41]. The reads from each species were counted by HTSeq-Count [42] and the differential expression determined by the R-based statistical DESeq [43].

Mapped genes were considered as differentially expressed genes (DEGs) if their relative gene expression levels showed an adjusted *p-*value (FDR) < 0.05 and an amplitude of differential expression of at least 4-FC (log2FC > 2.0 or < -2.0) between RKN samples and controls. Due to typically low levels of expression in plants, NBS–LRR genes were considered as differentially expressed genes with low fold-change (_L_DEG) [44], if their relative gene expression levels showed an adjusted *p-*value (FDR) < 0.05, regardless of the fold change (FC) amplitude of differential expression values. The visualization of commonly expressed genes among the libraries was conducted by UpSetR (https://gehlenborglab.shinyapps.io/upsetr/).

### Functional classification

Functional annotation and classification of the DEGs into categories via Gene Ontology (GO) terms was based on *A. duranensis* gene models annotation (http://peanutbase.org/). The Hypergeometric test for overrepresentation from FUNC package [45] was used to test for significantly enriched GO categories among *A. duranensis* DEGs using default parameters, and only genes with a FWER < 0.05 for overrepresentation were selected for further analysis. Transcription factors DEGs were identified based on the classification of the Plant TF database (http://planttfdb.cbi.pku.edu.cn/).

### MapMan ontology

To include NBS-LRR genes and obtain an as comprehensive as possible analysis [44], we used all *A. stenosperma* and *A. duranensis* differentially expressed genes with low fold-change (_L_DEG), which include all genes with significant differential relative gene expression between nematode inoculated and control samples at an adjusted *p-*value (FDR) < 0.05, regardless of its expression magnitude (FC). To identify their biological functions and involvement in biotic stress pathways, gene models from *A. duranensis* reference genome (https://peanutbase.org/) covered by the above selected RNA-Seq reads of *A. stenosperma* and *A. duranensis* were submitted to Mercator [46] against the *Arabidopsis thaliana* database, using default settings. The results were submitted to MapMan to visualize the expression of the genes in the biotic pathway [47].

### NBS-LRR physical clustering, expression profile and phylogenetic analysis

To identify physical gene clusters of NBS-LRR in *A. duranensis* reference genome, the definition of Richly et al., [48] was used; with two or more NBS-LRR genes occurring within a maximum of eight ORFs and less than 250 kb apart.

The *in silico* expression profile of these NBS-LRR genes was carried out by mapping the RNA-Seq data onto their previously predicted classification in the *A. duranensis* reference genome [26]. Those NBS-LRR genes differentially expressed with low fold-change (_L_DEG) were used to construct the expression clustering.

Clustering analysis of significant genes based on common expression patterns was conducted using the cutree function of gplots package from CRAN [49].

For the phylogenetic analysis of the *Arachis* NBS-LRR family, the NB-ARC domain (PF00931) was used for the HMMsearch against the 345 *A. duranensis* NBS-LRR predicted protein sequences described by Bertioli et al., [26]. Only the sequences with more than 50% of the full-length NB-ARC domain were kept and aligned using MAFFT software [50], using --auto parameter to select the best alignment strategy. We eliminated all columns of the alignment that contained more than 10% of gaps by using trimAl, to provide a more accurate alignment and construction of the phylogenetic tree [51]. Construction of the phylogenetic tree was conducted using RAxML software [52] with an automatic detection of the fittest evolutionary model and an estimated gamma distribution of rates of evolution. For bootstrap replicates, the ‘-autoMRE’ option to automatically stop RAxML upon convergence was used. Duplication analysis was conducted with McscanX (http://chibba.pgml.uga.edu/mcscan2/) as previously described for *A. duranensis* [29].

### Expression analysis by qRT-PCR

The expression analysis of candidate genes was conducted by qRT-PCR using inoculated samples gathered in pools and the respective Ctrl samples. For this, total RNA from three individuals at each collecting point (3, 6, and 9 DAI) were pooled at equal amounts to constitute a biological replicate of inoculated samples. Two independent replicates of inoculated (STR) and control samples were thus formed for each species (*A. stenosperma* and *A. duranensis*) and used for cDNA synthesis as described above.

Reactions were carried out using three technical replicates for each sample using the Platinum® SYBR® Green qPCR Super Mix-UDG w/ROX kit (Invitrogen, Carlsbad, CA, USA) according to manufacturer's recommendations on StepOne Plus Real-Time PCR System (Applied Biosystem Foster City, CA, USA). The qRT-PCR analysis and specific primer pairs design were conducted for 17 NBS-LRR genes (Additional file 1: Table S1) as previously described [14]. Average cycle threshold (Cq) values were estimated using the online real-time PCR Miner tool [53] and normalized to two reference genes (60S and ACT1), as previously established [39]. Expression ratios of transcripts from the inoculated pool relative to Ctrl pool were determined and statistically tested using REST 2009 software [54].

## Results

### RNA-Seq data for both *A. stenosperma* and *A. duranensis* mapped to the *A. duranensis* reference genome

On average, 97% of *A. duranensis* cleaned RNA-Seq reads from the three time-points studied could be mapped to the *A. duranensis* reference genome where they illuminated around 25,000 gene models (Table 1). In comparison, over 95% of the *A. stenosperma* cleaned reads were cross-mapped to the *A. duranensis* reference genome where they illuminated over 22,000 gene models. The intersection of the two datasets shows that 25,000 gene models are supported by both *A. duranensis* and *A. stenosperma* reads which represent 68% of the approximate 36,000 *A. duranensis* gene models [26] (Table 1). Hence, a large set of genes could be compared regarding differential expression between the two closely related *Arachis* species upon infection by *M. arenaria*.

**Table 1:** Statistics of *Arachis* spp. reads mapped onto the *A. duranensis* reference genome and analysis of the differentially expressed genes (DEGs) under RKN infection against the control

Library^a^	Number of mapped genes	% of mapped reads	Number of mapped genes	% of mapped reads	Number of _L_DEG (FDR < 0.05)	Number of DEG (FDR < 0.05) (Log2FC >2 or < -2)
	Replicate 1		Replicate 2			
DCTR	25,096	97.58	25,023	97.82	N/A	N/A
DN3	25,338	97.42	25,389	97.95	2,078	124
DN6	25,466	98.01	24,999	97.97	791	61
DN9	24,969	97.83	24,564	97.76	685	46
SCTR	22,914	96.39	22,375	96.89	N/A	N/A
SN3	21,602	95.4	22,237	96.37	472	79
SN6	21,337	94.81	21,914	95.88	2,314	472
SN9	22,193	96.39	21,615	96.31	479	128

^a^ Different time points after *M. arenaria* inoculation in *A. duranensis* DCTR – control; DN3 – 3 DAI; DN6 – 6 DAI; DN9 – 9 DAI; and *A. stenosperma* SCTR – control; SN3 – 3 DAI; SN6 – 6 DAI; SN9 9 DAI. N/A – Not Applied

### Different genes at different time points are regulated during upon nematode infection in the two *Arachis* species

**Fig. 1:**
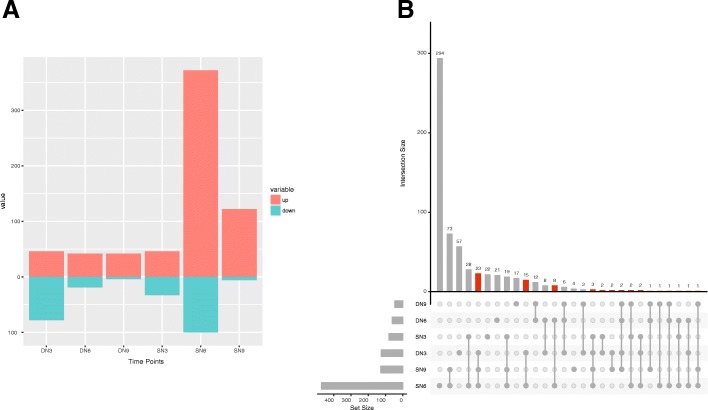
Differentially expressed genes (DEGs) in *A. duranensis* (DN) and *A. stenosperma* (SN) during *M. arenaria* infection at 3, 6 and 9 days after inoculation. **a** Barplot representing the number of DEGs up (red) and down (green) regulated at different time points of the analysis in *A. duranensis* (DN3, DN6, DN9) and *A. stenosperma* (SN3, SN6, SN9) infected roots. **b** Graphic representation of all the intersections between the DEGs in *A. duranensis* (DN3, DN6, DN9) and *A. stenosperma* (SN3, SN6, SN9) infected with *M. arenaria*. The red bars indicate the intersections between *A. duranensis* and *A. stenosperma*. The set size represents the number of DEGs at each condition (genotype/DAI) and the black dots, their intersections

We identified 189 and 657 DEGs after RKN infection in *A. duranensis* and *A. stenosperma* respectively, which showed statistical significance at adjusted *p-*value (FDR) <0.05 and two-fold change [log2 ratio of (control/stress) >2 or <-2]. For the highly resistant *A. stenosperma* most DEGs were modulated at 6 DAI (472) and least at 3 DAI (79), whereas in the moderately susceptible *A. duranensis* the majority of responsive genes occurred at 3 DAI (124), with a steady decrease in the numbers to 9 DAI (46) (Fig. 1a). This suggests that nematode resistance in *A. stenosperma* involves not only the modulation of a larger (3.5 fold) set of genes but also different and specific time points when compared to *A. duranensis*.

Only 63 DEGs were found to be shared between the two species (Fig. 1b) with the majority occurring between *A. duranensis* 3 DAI and *A. stenosperma* 6 DAI/9DAI (23), reinforcing distinct defense responses displayed by the two species against the nematode infection. Also, most exclusive genes appeared at the more relevant time point for the onset of the resistance response in each species with *A. stenosperma* harboring 294 exclusive genes at 6 DAI, while *A. duranensis* exhibited 57 unique genes at 3 DAI (Fig. 1b). Within *A. stenosperma*, the highest number of shared DEGs occurred between 6 and 9 DAI (73), followed by 3 and 6 DAI (28), indicating that the main specific molecular responses take place around the sixth day resulting in bursts of gene expression. In *A. duranensis*, most genes at 3 DAI are shared between the three time points and also with *A. stenosperma* at 6 and 9 DAI (23). Overall, the number of genes shared between all the time points in *A. stenosperma* is superior to that in *A. duranensis*, 19 and 6, respectively (Fig. 1b). This suggests that for the onset of the HR in the resistant species, a considerable number of genes must be triggered around the sixth post-infection day (6 DAI), with few remaining differentially expressed up to 9 DAI (Fig. 1b). There are no common DEGs across the three treatments and the two species.

The contrasting expression behavior of some genes in response to RKN between these two species (Fig. 1) was further corroborated by the distinct expression profiles of 14 candidate genes previously identified as candidates involved in *A. stenosperma* resistance to RKN [14, 16] (Additional file 2: Table S2). These genes are involved in pathogen perception, signal transduction, protein ubiquitination, hormone signaling and secondary metabolites production (*AsCHI2*, *AsGH3*, *AsTIR-NBS*, *AsBger*, *AsIOMT*, *AsKel*, *AsTAT*, *AsALKBH2*, *AsSLP*, *AsBTB*, *AsAraH8*, *AsTMV*, *AsPRR37* and *AsSAG*), which are crucial steps for the triggering and accomplishment of plant defense response. Accordingly, the majority of these genes showed not only upregulation in *A. stenosperma* (6 DAI), but also a significant downregulation in *A. duranensis* at 3 DAI (Additional file 3: Figure S1), reinforcing the relevance of these early time points and strengthening their roles in this defense response.

### The differentially expressed genes encompass different functional categories in the two *Arachis* species

**Fig. 2:**
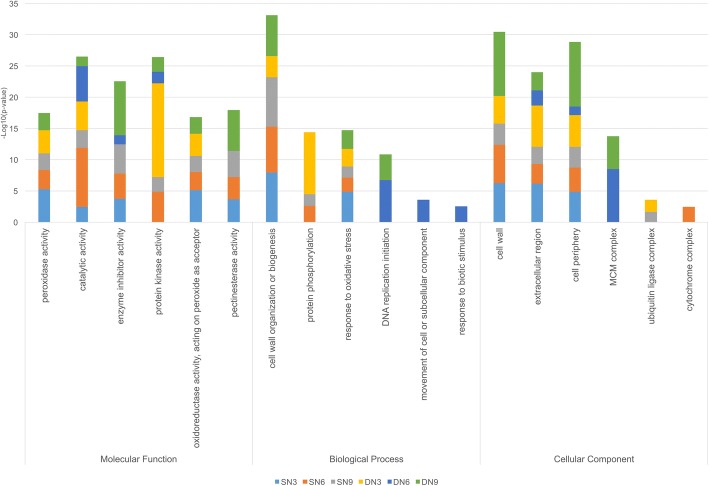
Enrichment of Gene Ontology (GO) terms in DEGs of *A. duranensis* (DN3, DN6, DN9) and *A. stenosperma* (SN3, SN6, SN9) roots infected with *M. arenaria* at different time points (3, 6, 9 DAI). DEGs are distributed in all three functional categories

Gene ontology (GO) was applied to categorize the function of DEGs identified in both species and to analyze the enrichment of these categories in each of the three time points after nematode inoculation (3, 6, 9 DAI) (Fig. 2). In the molecular function category, many GO terms related to catalytic activities, oxygen reduction and scavenging of ROS products such as peroxidases, oxireductases, pectinesterases and other antioxidant enzymes were significantly enriched in *A. stenosperma*, at 3 and 6 DAI, when the HR response occurs (Fig. 2) [55]. There is a clear enrichment for protein kinase activity in *A. duranensis* roots at 3 DAI when most genes are regulated in this genotype in response to the nematode. This is probably a result of intense cell activity, as kinases are known to regulate the majority of cellular pathways, especially those involved in signal transduction. In the biological process category, an enrichment of terms related to protein phosphorylation, compatible with kinase activity occurs at 3 DAI in *A. duranensis*, while the response to oxidative stress is a frequent GO term in *A. stenosperma*. Abundance of terms related to DNA replication and cellular components movement is also observed in the last two stages of *A. duranensis* interaction (6 and 9 DAI) suggesting the beginning of feeding sites formation (Fig. 2).

There was also enrichment for GO terms corresponding to localization or activity in the cell wall, inner cell membrane, and cell periphery. In *A. duranensis* at 6 and 9 DAI, there was an enrichment of GO terms corresponding to minichromosome maintenance complex (MCM), which is involved in both the initiation and the elongation step of eukaryotic DNA replication. Because of its role in genome duplication in proliferating cells, deregulation of the MCM function can result in chromosomal defects that may contribute to tumorigenesis. This coincides with the initial phase of cell gall formation in the moderately susceptible *A. duranensis* [15] and was not identified in *A. stenosperma* (Fig. 2).

### RKN infection triggers expression of pathogen-defense related pathways

**Fig. 3:**
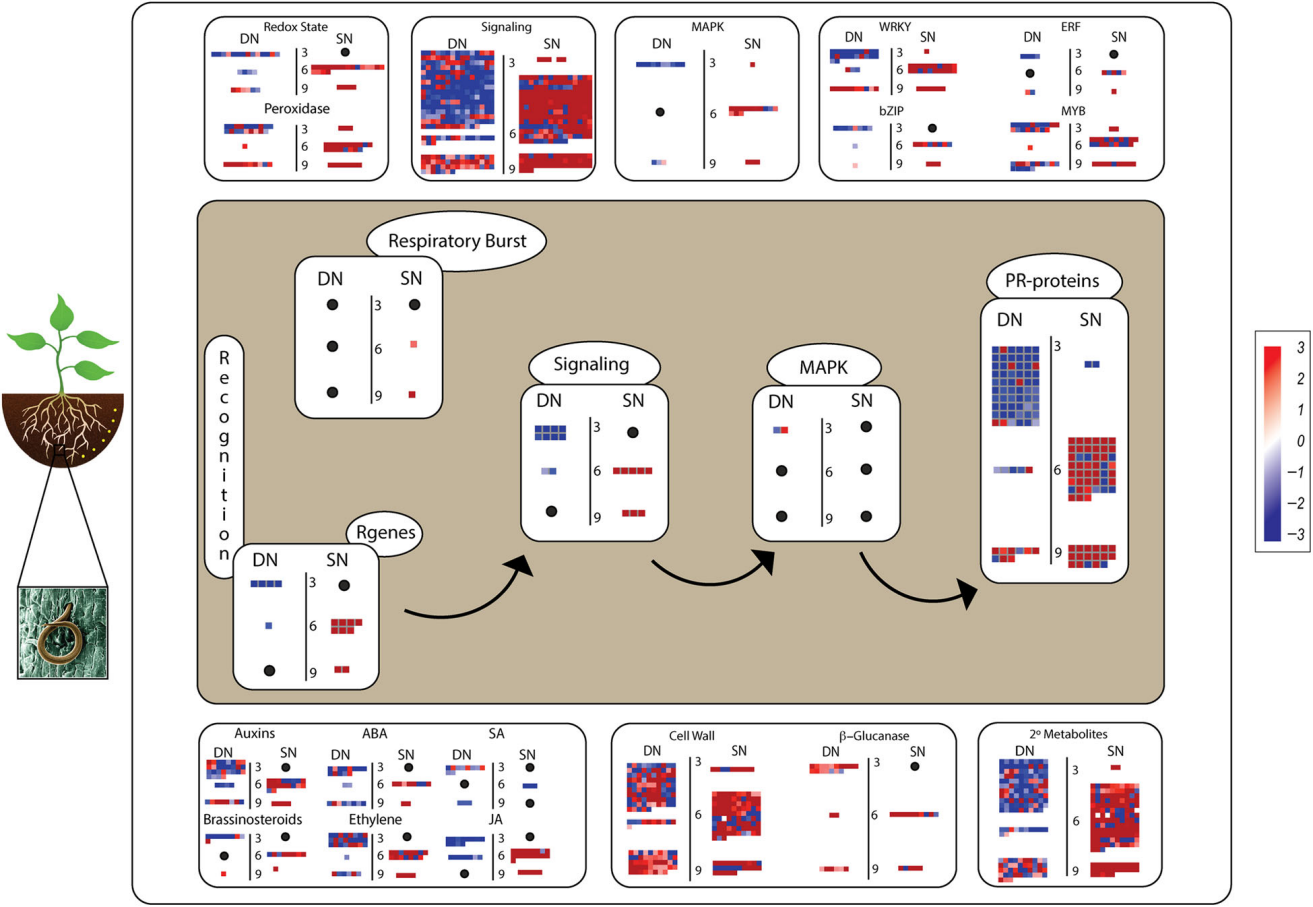
Graphical representation of metabolic pathways including biological functions assigned to _L_DEG (FDR < 0.05) in *A. duranensis* (DN) and *A. stenosperma* (SN) infected with *M. arenaria* at three different time points (3, 6 and 9 days after inoculation). The color scale represents differential gene expression magnitude (log_2_FC). Black dots indicate the lack of _L_DEG representatives

In the depiction “biotic response to stress”, responsive genes from both species belonging to families known to be involved in plant defense towards nematode infection (R genes, PR-proteins, secondary metabolites) were identified (Fig. 3). These genes were distributed in two main pathways (central and laterals) (Fig. 3), which include genes modulated in response to RKN mechanism of parasitism. First, as RKN penetrate the host roots, they cause mechanical wounding which initiates Jasmonic Acid (JA) and Ethylene (ET) production, leading to the activation of signaling molecules, Transcription Factors (TFs), cell wall enzymes, and secondary metabolites, as shown in both lateral sides of Fig. 3. Then, as RKN produce elicitors to establish the feeding sites, they also trigger a specific defense response, initiated by R genes and mediated by signaling molecules, including kinases (MAPK), culminating with the production of PRs and other defense proteins, as visualized in the central region of the MapMan illustration (Fig. 3).

The distribution of transcripts (_L_DEG) into defense pathways showed clearly the differences between the expression patterns of suites of genes activated in two contrasting species (Fig. 3). We theorize that in the resistant *A. stenosperma*, the immune response might be triggered by proteins that recognize specific effectors, such as resistance (R) proteins, as R genes seem to be most upregulated at 6 DAI and to a lesser extent at 9 DAI (Fig. 3). This is in clear contrast with *A. duranensis* transcripts assigned to the same function, that showed a general downregulation at 3 and 6 DAI. Following R genes activation, a respiratory burst and an enrichment of signaling molecules were observed mainly in *A. stenosperma* which might contribute to the control of the pathogen spread, leading to a striking rise in the expression of defense proteins (PRs) at 6 DAI, which coincides with HR in this species [55] (Fig. 3).

Pathogen-related proteins (PRs) were detected as the most represented defense protein, with 91 representatives modulated in both species, albeit with opposite expression trends. In *A. duranensis* 33 exclusive PR genes were mostly downregulated (3 DAI), while 21 unique to *A. stenosperma* were predominantly upregulated (6 DAI). Transcripts associated with pathogen cell wall breakdown (ß-glucanases), plant hormonal balance and cell wall modification and production of secondary metabolites were also differentially regulated in these two species (Fig. 3).

It is notable that the major signaling pathways triggered in *A. stenosperma* in response to *M. arenaria* seems to involve JA and ET, especially at 6 DAI (Fig. 3), although other hormones involved in plant development, such as auxins, abscisic acid (ABA), and brassinosteroids, also seem to participate in this defense response. In contrast, the activation of the Salicylic Acid (SA) signaling pathway seems to be more prevalent in *A. duranensis* than in *A. stenosperma*, which might be related to the well described jasmonate-salicylate antagonism occurring in other plant-pathogen interactions [56].

### Transcription factors induced upon RKN infection

In the large-scale transcriptional reprogramming observed in both species in response to RKN infection, four TF families WRKY, MYB, ERF and bZIP played a critical role (Fig. 3). Therefore, a more detailed expression analysis of 105 DEGs belonging to these families was conducted. Overall, the expression of these TFs varied during nematode infection according to the species and time point observed, with most genes showing a contrasting expression behavior between the two species (Additional file 4: Figure S2).

A subgroup of 11 representatives of the WRKY family showed upregulation in *A. stenosperma*, especially at 6 DAI, while downregulation in *A. duranensis*, mainly at 3 DAI (Additional file 4: Figure S2A), which suggests they might play a role in their contrasting resistance response. This is expected, as JA and SA plant defense pathways activated in this interaction, require large scale transcriptional reprogramming, including *WRKY* genes [57]. Likewise, four *MYB* genes showed strong upregulation in *A. stenosperma* at 6 DAI and downregulation in *A. duranensis* at 3 DAI (Additional file 4: Figure S2B). Members of *MYB* family are modulated by wounding [58], and in turn regulate some flavonoid genes involved in plant defense response, as observed in this study.

Five *bZIP* representatives showed a contrasting expression behavior in the two species studies, being upregulated in *A. stenosperma* mainly at 3 and 6 DAI and downregulated in *A. duranensis* at 3 DAI [59] (Additional file 4: Figure S2C). The same trend was observed amongst *ERF* members that are responsive to salt, cold, drought, wounding and fungi [60] and were, in their majority, strongly upregulated in *A. stenosperma* and downregulated in *A. duranensis* (Additional file 4: Figure S2D).

### NBS-LRR expression profiling upon RKN infection

The overall transcriptional reprogramming outline of transcripts associated to *A. stenosperma* resistance to RKN (Fig. 3) and the occurrence of the HR response strongly suggests that R genes, especially those encoding NBS-LRR proteins, play a pivotal importance in this incompatible interaction. Therefore, they were further characterized in this study in terms of expression behavior, phylogeny and physical clustering aiming to dissect their functional roles in the RKN response in both *Arachis* species.

From the 345 NBS-LRR-encoding genes of *A. duranensis* [26], 52 were differentially expressed genes with low fold-change (_L_DEG) (FDR<0.05) in at least one condition (genotype/DAI). Among these, 27 were modulated between infected and control roots in *A. stenosperma* and 37 in *A. duranensis*, of which only 12 were identified in both species.

**Fig. 4:**
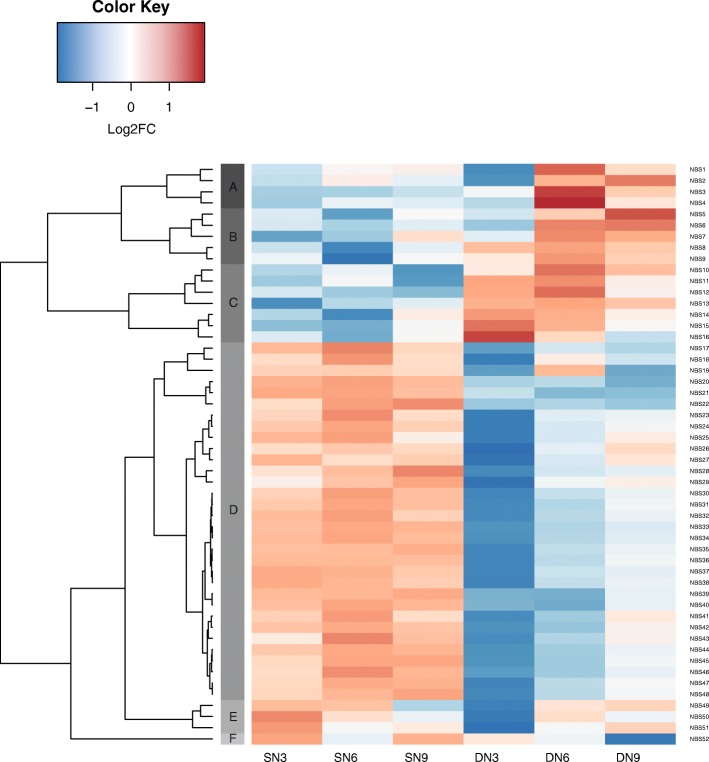
Heatmap of the *in silico* expression patterns of 52 NBS-LRR (_L_DEG) at different time points, in *A. stenosperma* (SN3, SN6 and SN9) and *A. duranensis* (DN3, DN6 and DN9) *M. arenaria* inoculated roots. The color key represents differential gene expression magnitude (log_2_FC)

Most of these 52 NBS-LRR genes showed a contrasting expression profile between A. duranensis and A. stenosperma upon nematode infection, with six expression clusters identified (Fig. 4). Genes comprising clusters A, B and C tend to show downregulation in *A. stenosperma* and upregulation in *A. duranensis*, whilst those in cluster D, E and F showed opposite expression profile. Cluster D comprised all the NBS-LRR that were upregulated in *A. stenosperma* at 6 DAI and most of those downregulated in *A. duranensis* at 3 DAI, which are the critical points of the onset of the defense response in each species [15].

In *A. duranensis*, most NBS-LRR genes were modulated at 3 DAI (34) with 20 exclusives to this time point (Additional file 5: Figure S3). On the other hand, in *A. stenosperma*, 6 DAI was the time point with most representatives (25) with 12 exclusives genes modulated (Additional file 5: Figure S3; Fig. 4). All the genes that are differentially regulated in both, *A. stenosperma* and *A. duranensis* are in the cluster D (Fig. 4). We suggest that the contrasting expression behaviour of the majority of NBS-LRR genes between the two species (Fig. 4) contribute to their distinctive defense responses, as *A. stenosperma* shows a strong Hypersensitive Response (HR), being practically immune to the nematode, whilst *A. duranensis* response propels a delay in the parasite penetration and development of the feeding cell [15].

### Chromosomal distribution of NBS-LRRs

The 37 NBS-LRR genes identified as _L_DEG in *A. duranensis* were distributed in all chromosomes, except for chromosome Aradu.A07 (http://peanutbase.org/), while the 27 *A. stenosperma* NBS-LRR (_L_DEG) were placed on fewer chromosomes with no representatives on chromosomes Aradu.A04, A06 and A07 (Additional file 6: Table S3).

All *Arachis* NBS-LRR (_L_DEG) were divided into two classes according to the presence or absence of the TOLL/interleukin-1 receptor (TIR), being named TIR-NBS-LRR and CC-NBS-LRR [19] and further classified into four subfamilies based on the presence of the above domains alone or in combination (N, TNL, TN, NL) [19]. Among the 37 *A. duranensis* NBS-LRR (_L_DEG), the great majority (92%) belonged to NL and TNL subclasses, whilst only three to TN (8.1%). A similar distribution of the 27 *A. stenosperma* NBS-LRR (_L_DEG) occurred with 15 in the NL subclass (55.5%), eight in TNL (29.6%) and only four TN (14.8%) (Additional file 7: Figure S4). Interestingly, no representatives of subclass N were identified as differentially expressed in response to RKN infection in neither of the two *Arachis* species studied.

### NBS-LRR phylogenetic analysis

**Fig. 5:**
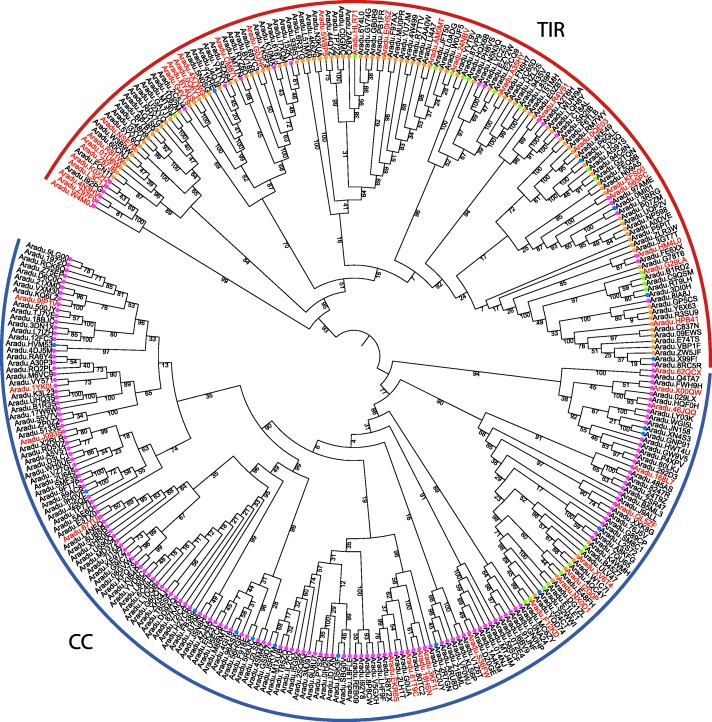
Phylogenetic tree showing the distribution of the NBS-LRR genes of *A. duranensis*. The NBS-LRR subclasses are represented by colored dots as follows: TNL (orange), TNx (green), xNL (pink) and xNx (blue). The names of the 52 _L_DEG (FDR<0.05) genes are represented in red

For the phylogenetic analysis, we kept only the 314 *A. duranensis* proteins that had more than 50% of the full-length NB-ARC domain (PF00931). The midpoint-rooted phylogenetic tree exhibited a basal separation into two major groups supported by high bootstrap values (93) (Fig. 5). The first group was mainly composed of TIR-type NBS-LRRs, whilst the second of CC-type NBS-LRRs. (Fig. 5). A small monophyletic group of CC-type NBS-LRRs, supported by high bootstrap value (99) was outgroup and more closely related to the TIR-types than to the rest of the CC-types. This suggest that the CC-type might be the ancestral NBS-LRR structure and that the TIR-type evolved secondarily by losing the CC domain and gaining a TIR domain. We also noted that in the clade containing most of CC-types, a few proteins with both a TIR and a CC domain were observed (Fig. 5; green and orange circles). Interestingly, all these cases belong to one single monophyletic, albeit not highly supported clade (bootstrap=23). This suggests that a subgroup of CC-type NBS-LRRs might have secondarily gained a TIR domain.

Among the 314 *A. duranensis* corresponding genes, 46 were identified as _L_DEGs and are highlighted in the phylogenetic tree (colored names - Fig. 5). These genes were distributed across different branches of the tree, showing that their expression was not restricted to a specific subfamily of NBS-LRR or even to a CC or TIR type. However, genes located in the same chromosome tend to group in subclades in the phylogenetic tree. This is consistent with tandem duplication being the most common type of duplication (46% of duplicated NBS-LRR genes), while proximal and dispersed duplication represented only 28 and 21%, respectively (Additional file 6: Table S3). The duplication pattern did not correlate with the NBS-type, being equally distributed between the CC and TIR-types. Genes not harboring a sufficiently complete NB-ARC domain were not integrated in this tree; however, they showed a similar expression pattern to the rest of their paralogous copies (Figs. 4 and 5).

### NBS-LRR genes physical clustering

**Fig. 6:**
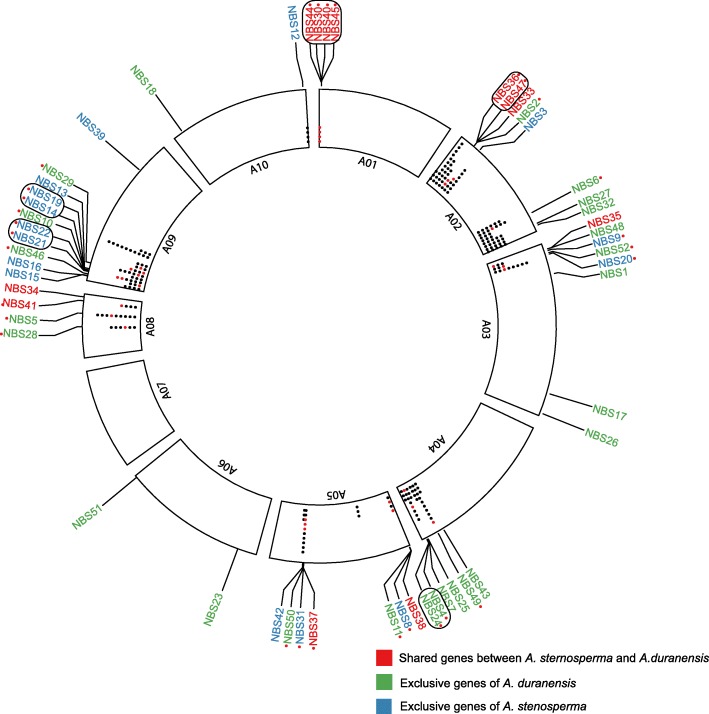
Distribution of *A. stenosperma* and *A. duranensis* NBS-LRR genes in clusters, on the *A. duranensis* chromosomes. Black dots show NBS-LLR genes in clusters; red dots show _L_DEGs in clusters. Genes shared between *A. duranensis* and *A. stenosperma* are named in red, *A. stenosperma* exclusive genes in blue and *A. duranensis*, in green. Red dots next to gene names highlight the _L_DEGs. Physical clusters containing _L_DEGs in pairs from the same species are circled

From all NBS-LRR genes identified, 55% (172) were in clusters located on all *A. duranensis* chromosomes (except for A06 and A07), which are represented by dots inside the chromosomes in Fig. 6. From these, 29 were _L_DEGs (red dots) with no representatives in chromosomes Aradu.A06, A07 and A10. (Figure 6). The majority of these NBS-LRR clusters contained only one _L_DEG representative; however, in chromosomes Aradu.A01, A02, A04, and A09 some of these low expressed genes (_L_DEG) (12) appeared in pairs and belonged to the same species. All these five clusters containing the _L_DEG are homogeneous, where the same type of NBS (CC or TIR) is observed, with three clusters assigned to TIR-type and two to CC-type (Fig. 6). Homogeneous clusters are expected due to the predominant type of duplication being tandem.

In chromosomes Aradu.A01 and A02, we identified NBS-LRR clusters constituted respectively of four and two _L_DEGs in both *A. stenosperma* and *A. duranensis* (Fig. 6). These six genes belong to the expression group D (Fig. 4), characterized by a strong upregulation in *A. stenosperma* (6 DAI) and downregulation in *A. duranensis* (3 DAI) (Figs. 4 and 6). Another cluster containing two _L_DEGs was found in chromosome Aradu.A04. However, these genes appeared exclusively in *A. duranensis* and belonged to different expression groups (A and D) with opposite expression trends upon nematode infection (Figs. 4 and 6).

Chromosome Aradu.A09 harbored the largest number of differentially expressed genes with low fold-change (_L_DEG) NBS-LRRs in physical clusters (Fig. 6), and contained two NBS clusters with genes exclusively regulated in *A. stenosperma*. Fittingly, a significant QTL for *M. arenaria* resistance has been found in this chromosome [12]. Moreover, most of the genes in these two clusters (except by NBS14) belong to expression cluster D, which showed denotable upregulation in *A. stenosperma* (6 DAI) (Fig. 4).

All the NBS-LRR (_L_DEG) grouped in clusters were compared against the Plant Resistance Genes database PRGdb (http://prgdb.crg.eu) using BlastP to verify their homology to known resistance genes. Remarkably, four genes in chromosome Aradu.A09 (NBS22, NBS14, NBS19 and the non-_L_DEG Aradu.Y6X63), which were exclusively regulated in *A. stenosperma*, showed homology to the resistance gene *Gro*1-4 against the yellow potato cyst nematode *Globodera rostochiensis* [61]. In contrast, NBS-LRR (_L_DEG) showing no contrasting expression behavior between the two species within the same clusters lacked homology to known nematode resistance genes, with higher similarity to resistance genes against virus or fungi.

### Expression analysis by qRT-PCR

**Fig. 7:**
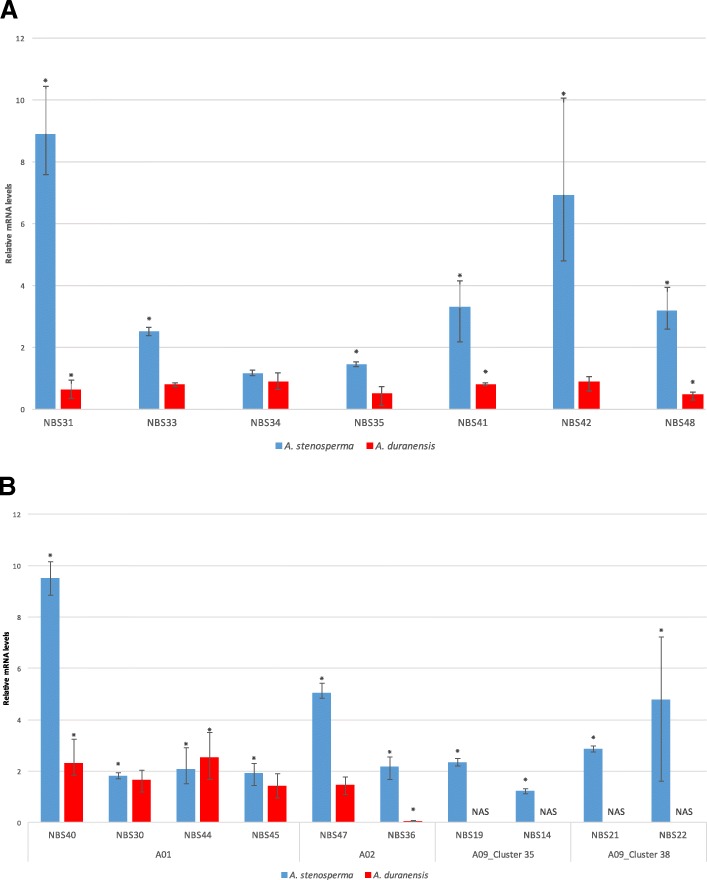
Relative mRNA levels of the NBS-LRR genes in *A. duranensis* and *A. stenosperma*. **a** Transcripts relative quantification of ten _L_DEGs of *A. duranensis* (red) and *A. stenosperma* (blue) distributed in four clusters, on chromosomes Aradu.A01, A02, and A09. The expression levels of the _L_DEGs in *A. duranensis* and *A. stenosperma* relative to control. Non-amplified sequences (NAS) corresponding to exclusive genes of *A. stenosperma*. **b** Expression levels of seven _L_DEGs in *A. stenosperma* (blue) and *A. duranensis* (red) inoculated with *M. arenaria* relative to control samples. Statistically significant regulated genes (*) and error bars

The expression behavior of seven representatives of the major NBS-LRR expression group (D) (Fig. 4) was validated in both *Arachis* species by qRT-PCR using specific primers (Additional file 1: Table S1) and RNA pooled from the three time points for both species. Expression analysis showed a contrasting behavior between the two species for all NBS-LRR transcripts tested (Fig. 7a) in accordance with *in silico* analysis (Fig. 4), i.e., an upregulation (ranging from 1.168 to 8.89 FC) in *A. stenosperma* and downregulation (ranging from 0.883 to 0.466 FC) in *A. duranensis* (Fig. 7a).

The expression profiles of ten NBS-LRR (_L_DEGs) genes located in clusters (Fig. 6) was also evaluated by qRT-PCR using specific primers (Additional file 1: Table S1) and RNA pooled from the three time points studied in *A. duranensis* and *A. stenosperma* (Fig. 7b). The qRT-PCR analysis corroborated the induction of all the _L_DEGs belonging to clusters on chromosomes Aradu.A01, A02 and A09 in *A. stenosperma*. Nevertheless, in the moderately susceptible species (*A. duranensis*) only six genes were amplified, as four _L_DEGs were *A. stenosperma* exclusive (NAS) (Fig. 7b).

## Discussion

The genomic similarity between *A. stenosperma* and *A. duranensis* allowed the mapping of 95% of *A. stenosperma* RNA-Seq reads, which lacks a complete sequenced genome, to the *A. duranensis* reference genome with no significant loss of information, as already seen for other related species [62–64]. We identified over 26% *A. stenosperma* genes as significantly differently expressed (FDR < 0.05) with at least four fold (DEGs) when infected by RKN, compared to control in one or more time points. The smaller number of DEGs found, in comparison to our previous works using *de novo* assembly [16], is probably due to a less redundant, albeit more comprehensive, reference gene set coming from the genome assembly. Genome-based strategies are known to provide less chimeric contigs, better filtration of contaminations and a greater representation of the low-abundant transcripts [35].

According to Proite et al., [15], the development of *M. arenaria* in the resistant (*A. stenosperma*) and in the moderately susceptible (*A. duranensis*) differed along the interaction. In the resistant genotype, no giant cells were formed and the HR response was triggered, blocking the nematode development, while in the moderately susceptible species, the giant cell were formed around the 19th day after the inoculation (J3 to J4), albeit in smaller numbers. Likewise, in this study, a distinct downstream gene reprogramming upon nematode infection was observed in the two *Arachis* species, with most genes being upregulated in the resistant, whilst downregulated in the more susceptible species. The timing of gene expression responses was also different, as in *A. duranensis* most DEGs appear at the very beginning of the interaction (3 DAI) which is compatible to the early onset of the PTI (Pattern-Triggered Immunity) [65–67], whilst in *A. stenosperma* most genes are upregulated at 6 DAI, resembling an ETI (Effector-Triggered Immunity) type of response. In addition, a higher number of upregulated genes in *A. stenosperma* assigned to the categories of R genes, PR proteins, TFs and secondary metabolites were upregulated at 6 and 9 DAI. These genes expression patterns coincide with the occurrence of the Hypersensitive Response (HR) displayed by this genotype [15]. The distinctive expression behavior of candidate genes previously identified as being involved in HR response to *M. arenaria* [16] herein observed between the two *Arachis* species, corroborates their different mechanisms of defense responses.

Despite JA being classically more related to resistance to necrotrophic and SA to biotrophic pathogens, Jasmonate–Ethylene (ET/JA) pathways have also been found to be essential to the resistance to some biotrophic pathogens [68–70]. In *A. stenosperma*, a clear upregulation of genes involved in the ET/JA pathways was observed, particularly at 6 and 9 DAI, while genes related to salicylic acid biosynthesis were mostly downregulated. Although a reciprocal inhibition between SA and JA is well documented [71, 72], many examples show that the interplay between these hormones pathways is not always antagonistic [73, 74], with some evidences that SA can induce JA synthesis and promote ETI responses [75]. This seems to be the case here, with the suppression of SA possibly being triggered by the enrichment for other genes involved in the ET/JA pathways, such as auxins induced by the RKN nematode [76].

Typically, resistance to RKN in both wild and domesticated plants is conferred by R genes mostly from the NBS-LRR family, which sense nematode effectors and trigger the first specific response (ETI) to the nematode infection initiating a cascade of defense genes [77]. However, a typical plant genome contains hundreds of NBS-LRR genes, located in chromosomal clusters, which makes the dissection of the targeted locus and its isolation by functional or genetic recombination rather difficult [78]. In this study, the analysis of NBS-LRR genes expression in contrasting genotypes enabled the identification of 27 differentially expressed genes with low fold-change (_L_DEG) in *A. stenosperma* RKN infected roots, of which 19 showed upregulation, while their orthologs in *A. duranensis* were downregulated (Fig. 4), pointing to their involvement in the HR response displayed by the resistant species. The phylogenetic analysis of the NBS-LRR family shows a basal separation in two highly supported clades, one mainly composed of TIR-type and the other of CC-type. This corroborates previous studies in *A. duranensis* [26, 27] and other plants species [79, 80]. However, the different phylogenetic groups do not match the different NBS-LRRs subclasses that are based on the arrangement of the different main domains (CC, TIR, NBS, LRR). As our phylogenetic tree is based on the widely conserved NB-ARC domain, this strongly suggests promiscuous domain shuffling in the NBS-LRR family, resulting in multiple convergent emergences of the same arrangements rather than common ancestral inherited arrangements.

The occurrence of physical clusters of NBS-LRR genes has been reported in different plant species, including *A. duranensis* [26, 27] and can be associated to the co-expression of these genes, the type of duplication that occurred during evolution of the plant species or their biological role [81–83]. In our study, most of the NBS–LRR (_L_DEG) belonged to the tandem duplication type in both *Arachis* species. This kind of duplication is well known to contribute to the expansion of genes involved in stress responses such as those responding to pathogens [27, 84, 85]. Also, as seen in other studies [26, 86], most NBS-LRR clusters found here are accumulated in the hot recombination regions of the distal chromosomal regions, which favor recombination reshuffling and R loci which require rapid diversification to overcome the emergence of new pathogen races.

Over a third (32%) of the NBS-LRRs responding to *M. arenaria* infection in both species were found in pairs with the same expression pattern within these clusters, which is often necessary for an effective resistance [87]. Clustering has frequently been related to co-expression of functionally related genes [23, 85, 88], and suitably, a larger number of these NBS-LRR pairs occurred in the resistant species (*A. stenosperma*) than in the more susceptible species (*A. duranensis*).

Remarkably, on chromosome 9, two NBS-LRR clusters are composed exclusively of *A. stenosperma*_L_DEGs. Among them, three genes (NBS14, NBS19 and NBS22) show high similarity to the potato *Gro1-4* gene, which confers resistance to another endoparasitic nematode, the cyst nematode *G. rostochiensis* [61], and belong to the TIR-type class which is often associated with resistance to different types of biotic stresses [27, 89, 90]. Also on chromosome Aradu.A02, another two NBS-LRRs (NBS36 and NBS47) showed high similarity to *Gro1-4*, and although belonging to gene clusters shared between both *Arachis* species, they exhibited contrasting gene expression behavior. Some genes in this same cluster with no significant differential expression were assigned to other R-gene categories, showing that, albeit genes in a cluster are physically close, they might not all show significant differential expression or bear the same function [23].

Despite differences on the parasitic process and feeding site structures between RKN and the cyst nematode *Globodera* spp. [91], both are obligate sedentary endoparasites, relying on juveniles (J2) to penetrate the roots and induce feeding sites. Therefore, the similarity between the NBS-LRRs identified here, especially the RKN-responsive in *A. stenosperma*, and known nematode R genes, might pose a functional role for these genes in *Arachis*. In addition, the fact that these NBS-LRR clusters are located on chromosome Aradu.A02 and A09, near two major QTLs identified in a population derived from a cross between the above species, which are associated with the reduction of *M. arenaria* root galling and egg production [12], reinforces their role in this strong resistance response. Thus, the genes located in the above clusters will be prioritized for further functional investigation, either by their overexpression in susceptible genotypes or genome editing.

Plant immune signaling network is rather complex. Nonetheless, this complexity is necessary, as pathogens continuously evolve a new repertoire to overcome host immune responses and plant adaptation is much slower than pathogen evolution [92]. Therefore, plants should carefully balance this complex network, as it might have a negative impact on their fitness. The use of contrasting wild *Arachis* species with different levels of resistance to *M. arenaria* has enabled the identification of expressed NBS-LRR clusters containing genes with potential functional relevance in this defense response, reinforcing the usefulness of comparative genomic analysis of NBS genes as an efficient means of mining functional R genes. Also, studies showing that rapidly evolved NBS genes from different species are capable of conferring defense against the same pathogen in *Gramineae* [93], strengthening their use in breeding programs.

## Conclusion

The further isolation and characterization of the R genes responsible for triggering effective defense cascades found in this study and their careful selection and stacking in order to balance the strength and specificity of this immune response, can contribute for a more durable resistance in peanut and other RKN affected crops.

## Additional files

Additional file 1: **Table S1.** Primers used for qRT-PCR analysis. (XLSX 11 kb)

Additional file 2: **Table S2.** *Arachis stenosperma* defense candidate genes against the RKN *M. arenaria*. (XLSX 27 kb)

Additional file 3: **Figure S1.** Expression profiles of 14 nematode responsive candidate genes in *A. stenosperma* and *A. duranensis* at 3,6 and 9 DAI with *M. arenaria*. (PDF 46 kb)

Additional file 4: **Figure S2.** Transcriptional profile of members of four TF families: **(A)** WRKY **(B)** MYB **(C)** bZIP **(D)** ERF in *A. stenosperma* (SN3, SN6, SN9) and *A. duranensis* (DN3, DN6, DN9) in response to RKN infection. (PDF 1356 kb)

Additional file 5: **Figure S3.** Intersections between the NBS-LRR (_L_DEG) (FDR<0.05) in *A. duranensis* (DN3, DN6, DN9) and *A. stenosperma* (SN3, SN6, SN9) infected with *M. arenaria*. The set size represents the number of RKN- responsive genes in each condition (genotype/DAI) and the black dots their intersections. (PDF 8 kb)

Additional file 6: **Table S3.**_L_DEG NBS-LRR genes (FDR < 0.05) in *Arachis duranensis* and *Arachis stenosperma*. (XLSX 34 kb)

Additional file 7: **Figure S4.** Distribution of *A. stenosperma* and *A. duranensis* expressed NBS-LRR subclasses in *A. duranensis* chromosomes (http://peanutbase.org/). (PDF 1178 kb)

## Abbreviations

CC: Coiled-Coil
DAI: Days After Inoculation
DEG: Differentially Expressed Genes
FC: Fold-Change
FDR: False Discovery Rate
GO: Gene Ontology
HR: Hypersensitive Response
LRR: Leucine Rich Repeats
NBS: Nucleotide Binding Site
qRT-PCR: quantitative Reverse Transcription-Polymerase Chain Reaction
QTL: Quantitative Trait Locus
RGA: Resistance gene analogs
RKN: Root-Knot nematode
TF: Transcription Factors
TIR: Toll/interleukin receptor

## References

[1] Dong W, Holbrook CC, Timper P, Brenneman TB, Mullinix BG (2007). Comparison of methods for assessing resistance to *Meloidogyne arenaria* in peanut. J Nematol.

[2] Nelson SC, Simpson CE, Starr JL (1989). Resistance to *Meloidogyne arenaria* in *Arachis* spp. germplasm. J Nematol.

[3] Dwivedi SL, Bertioli DJ, Crouch JH, Valls JF, Upadhyaya HD, Fárvero A, Kole C, Genome mapping and molecular breeding in plants (2007). Peanut. Oilseeds.

[4] Stalker HT, Moss JP (1987). Cytogenetics and utilization of *Arachis* Species. Adv Agron.

[5] Stalker HT, Tallury SP, Ozias-Akins P, Bertioli D, Leal-Bertioli SCM (2013). The value of diploid peanut relatives for breeding and genomics. Peanut Sci.

[6] Chu Y, Gill R, Timper P, Holbrook CC, Ozias-Akins P (2016). Identification of rare recombinants leads to tightly linked markers for nematode resistance in peanut. Peanut Sci.

[7] Clevenger J, Chu Y, Arrais Guimaraes L, Maia T, Bertioli D, Leal-Bertioli S (2017). Gene expression profiling describes the genetic regulation of *Meloidogyne arenaria* resistance in *Arachis hypogaea* and reveals a candidate gene for resistance. Sci Rep.

[8] Nagy ED, Chu Y, Guo Y, Khanal S, Tang S, Li Y (2010). Recombination is suppressed in an alien introgression on chromosome 5A of peanut harboring Rma, a dominant root-knot nematode resistance gene. Mol Breed.

[9] Simpson CE, Starr JL (2001). Registration of “COAN” peanut. Crop Sci.

[10] Guimarães PM, Brasileiro ACM, Morgante CV, Martins ACQ, Pappas G, Silva OB (2012). Global transcriptome analysis of two wild relatives of peanut under drought and fungi infection. BMC Genomics.

[11] Leal-Bertioli SCMDM, De Farias MP, Silva PÍTIT, Guimarães PM, Brasileiro ACM, Bertioli DJ (2010). Ultrastructure of the initial interaction of *Puccinia arachidis* and *Cercosporidium personatum* with leaves of *Arachis hypogaea* and *Arachis stenosperma*. J Phytopathol.

[12] Leal-Bertioli SCM, Moretzsohn MC, Roberts PA, Ballén-Taborda C, Borba TCO, Valdisser PA (2016). Genetic mapping of resistance to *Meloidogyne arenaria* in *Arachis stenosperma*: a new source of nematode resistance for peanut. G3: Genes, Genomes. Genetics.

[13] Michelotto MD, Barioni W, De Resende MDV, De Godoy IJ, Leonardecz E, Fávero AP (2015). Identification of fungus resistant wild accessions and interspecific hybrids of the genus *Arachis*. PLoS One.

[14] Morgante CV, Brasileiro ACM, Roberts PA, Guimaraes LA, Araujo ACG, Fonseca LN (2013). A survey of genes involved in *Arachis stenosperma* resistance to *Meloidogyne arenaria* race 1. Funct Plant Biol.

[15] Proite K, Carneiro R, Falcao R, Gomes A, Leal-Bertioli S, Guimaraes P (2008). Post-infection development and histopathology of *Meloidogyne arenaria* race 1 on *Arachis* spp. Plant Pathol.

[16] Guimaraes PM, Guimaraes LA, Morgante CV, Silva OB, Araujo ACG, Martins ACQ (2015). Root transcriptome analysis of wild peanut reveals candidate genes for nematode resistance. PLoS One.

[17] Dangl JL, Horvath DM, Staskawicz BJ (2013). Pivoting the plant immune system from dissection to deployment. Science.

[18] Kourelis J, van der Hoorn RAL (2018). Defended to the nines: 25 years of resistance gene cloning identifies nine mechanisms for R protein function. Plant Cell.

[19] Meyers BC, Dickerman AW, Michelmore RW, Sivaramakrishnan S, Sobral BW, Young ND (1999). Plant disease resistance genes encode members of an ancient and diverse protein family within the nucleotide-binding superfamily. Plant J.

[20] Li R, Rashotte AM, Singh NK, Weaver DB, Lawrence KS, Locy RD (2015). Integrated signaling networks in plant responses to sedentary endoparasitic nematodes: a perspective. Plant Cell Rep.

[21] Barbary A, Djian-Caporalino C, Marteu N, Fazari A, Caromel B, Castagnone-Sereno P (2016). Plant genetic background increasing the efficiency and durability of major resistance genes to root-knot nematodes can be resolved into a few resistance QTLs. Front Plant Sci.

[22] Bertioli DJ, Leal-Bertioli SCM, Lion MB, Santos VL, Pappas G, Cannon SB (2003). A large scale analysis of resistance gene homologues in *Arachis*. Mol Gen Genomics.

[23] Ratnaparkhe MB, Wang X, Li J, Compton RO, Rainville LK, Lemke C (2011). Comparative analysis of peanut NBS-LRR gene clusters suggests evolutionary innovation among duplicated domains and erosion of gene microsynteny. New Phytol.

[24] Wang H, Penmetsa RV, Yuan M, Gong L, Zhao Y, Guo B (2012). Development and characterization of BAC-end sequence derived SSRs, and their incorporation into a new higher density genetic map for cultivated peanut (*Arachis hypogaea* L.). BMC Plant Biol.

[25] Yuksel B, Estill J, Schulze S, Paterson A (2005). Organization and evolution of resistance gene analogs in peanut. Mol Gen Genomics.

[26] Bertioli DJ, Cannon SB, Froenicke L, Huang G, Farmer AD, Cannon EKS (2016). The genome sequences of *Arachis duranensis* and *Arachis ipaensis*, the diploid ancestors of cultivated peanut. Nat Genet.

[27] Song H, Wang P, Li C, Han S, Zhao C, Xia H (2017). Comparative analysis of NBS-LRR genes and their response to *Aspergillus flavus* in *Arachis*. PLoS One.

[28] Zheng YX, Li CJ, Liu Y, Yan CX, Zhang TT, Zhuang WJ, Shan SH (2013). Cloning and characterization of an NBS-LRR resistance gene from peanuts (*Arachis hypogaea* L.). Physiol Mol Plant Pathol.

[29] Guimaraes LA, Mota APZ, Araujo ACG, de Alencar Figueiredo LF, Pereira BM, de Passos Saraiva MA (2017). Genome-wide analysis of expansin superfamily in wild *Arachis* discloses a stress-responsive expansin-like B gene. Plant Mol Biol.

[30] Wang P, Song H, Li C, Li P, Li A, Guan H (2017). Genome-wide dissection of the heat shock transcription factor family genes in *Arachis*. Front Plant Sci.

[31] Song H, Wang P, Lin J-Y, Zhao C, Bi Y, Wang X (2016). Genome-wide identification and characterization of WRKY gene family in peanut. Front Plant Sci.

[32] Berardini TZ, Reiser L, Li D, Mezheritsky Y, Muller R, Strait E (2015). The Arabidopsis information resource: Making and mining the “gold standard” annotated reference plant genome. Genesis.

[33] Cui J, Luan Y, Jiang N, Bao H, Meng J, Science L (2016). Comparative transcriptome analysis between resistant and susceptible tomato allows the identification of lncRNA16397 conferring resistance to *Phytophtora infestans* by co-expressing glutaredoxin. The Plant J.

[34] Draffehn AM, Li L, Krezdorn N, Ding J, Lübeck J, Strahwald J (2013). Comparative transcript profiling by SuperSAGE identifies novel candidate genes for controlling potato quantitative resistance to late blight not compromised by late maturity. Front Plant Sci.

[35] Jain S, Chittem K, Brueggeman R, Osorno JM, Richards J, Nelson BD (2016). Comparative transcriptome analysis of resistant and susceptible common bean genotypes in response to soybean cyst nematode infection. PLoS One.

[36] Li R, Rashotte AM, Singh NK, Lawrence KS, Weaver DB, Locy RD (2015). Transcriptome analysis of cotton (*Gossypium hirsutum* L.) genotypes that are susceptible, resistant, and hypersensitive to reniform nematode (*Rotylenchulus reniformis*). PLoS One.

[37] Xing X, Li X, Zhang M, Wang Y, Liu B, Xi Q (2016). Transcriptome analysis of resistant and susceptible tobacco (*Nicotiana tabacum*) in response to root-knot nematode *Meloidogyne incognita* infection. Biochem Biophys Res Commun.

[38] Wu J, Zhu J, Wang L, Wang S (2017). Genome-wide association study identifies NBS-LRR-encoding genes related with anthracnose and common bacterial blight in the common bean. Front Plant Sci.

[39] Morgante CV, Guimaraes PM, Martins A, Araujo ACG, Leal-Bertioli SCM, Bertioli DJ (2011). Reference genes for quantitative reverse transcription-polymerase chain reaction expression studies in wild and cultivated peanut. BMC Res Notes.

[40] Bolger AM, Lohse M, Usadel B (2014). Trimmomatic: a flexible trimmer for Illumina sequence data. Bioinformatics.

[41] Wu TD, Nacu S (2010). Fast and SNP-tolerant detection of complex variants and splicing in short reads. Bioinformatics.

[42] Anders S, McCarthy DJ, Chen Y, Okoniewski M, Smyth GK, Huber W (2013). Count-based differential expression analysis of RNA sequencing data using R and Bioconductor. Nat Protoc.

[43] Love M, Anders S, Huber W (2014). Differential analysis of count data–the DESeq2 package. Genome Biol.

[44] Jain N, Thatte J, Braciale T, Ley K, O’Connell M, Lee JK (2003). Local-pooled-error test for identifying differentially expressed genes with a small number of replicated microarrays. Bioinformatics.

[45] Prüfer K, Muetzel B, Do H-H, Weiss G, Khaitovich P, Rahm E (2007). FUNC: a package for detecting significant associations between gene sets and ontological annotations. BMC Bioinformatics.

[46] Lohse M, Nagel A, Herter T, May P, Schroda M, Zrenner R (2014). Mercator: a fast and simple web server for genome scale functional annotation of plant sequence data. Plant Cell Environ.

[47] Thimm O, Bläsing O, Gibon Y, Nagel A, Meyer S, Krüger P (2004). MAPMAN: A user-driven tool to display genomics data sets onto diagrams of metabolic pathways and other biological processes. Plant J.

[48] Richly E, Kurth J, Leister D (2002). Mode of amplification and reorganization of resistance genes during recent *Arabidopsis thaliana* evolution. Mol Biol Evol.

[49] Warnes GR, Bolker B, Bonebakker L, Gentleman R, Huber W, Liaw A (2015). gplots: Various R programming tools for plotting data. R Package version 2.16.0.

[50] Katoh K, Misawa K, Kuma K, Miyata T (2002). MAFFT: a novel method for rapid multiple sequence alignment based on fast Fourier transform. Nucleic Acids Res.

[51] Capella-Gutiérrez S, Silla-Martínez JM, Gabaldón T (2009). trimAl: a tool for automated alignment trimming in large-scale phylogenetic analyses. Bioinformatics.

[52] Stamatakis A (2006). RAxML-VI-HPC: maximum likelihood-based phylogenetic analyses with thousands of taxa and mixed models. Bioinformatics.

[53] Zhao S, Fernald RD (2005). Comprehensive algorithm for quantitative real-time polymerase chain reaction. J Comput Biol.

[54] Pfaffl MW, Horgan GW, Dempfle L (2002). Relative expression software tool (REST{©}) for group-wise comparison and statistical analysis of relative expression results in real-time PCR. Nucleic Acids Res.

[55] Proite K, Carneiro R, Falcão R, Gomes A, Leal-Bertioli S, Guimarães P (2008). Post-infection development and histopathology of *Meloidogyne arenaria* race 1 on *Arachis* spp. Plant Pathol.

[56] Kunkel BN, Brooks DM (2002). Cross talk between signaling pathways in pathogen defense. Curr Opin Plant Biol.

[57] Pandey SP, Somssich IE (2009). The role of WRKY transcription factors in plant immunity. Plant Physiol.

[58] Cheong YH, Chang H-SS, Gupta R, Wang X, Zhu T, Luan S (2002). Transcriptional profiling reveals novel interactions between wounding, pathogen, abiotic stress, and hormonal responses in Arabidopsis. Plant Physiol.

[59] Hwang I, Manoharan RK, Kang J, Chung M, Kim Y, Nou I. Genome-wide identification and characterization of bZIP transcription factors in *Brassica oleracea* under cold stress. Biomed Res Int. 2016; 10.1155/2016/4376598. 10.1155/2016/4376598 PMC4893578 27314020

[60] Jisha V, Dampanaboina L, Vadassery J, Mithöfer A, Kappara S, Ramanan R (2015). Overexpression of an AP2/ERF type transcription factor OsEREBP1 confers biotic and abiotic stress tolerance in rice. PLoS One.

[61] Paal J, Henselewski H, Muth J, Meksem K, Menéndez CM, Salamini F (2004). Molecular cloning of the potato Gro1-4 gene conferring resistance to pathotype Ro1 of the root cyst nematode *Globodera rostochiensis*, based on a candidate gene approach. Plant J.

[62] Collins LJ, Biggs PJ, Voelckel C, Joly S (2008). An approach to transcriptome analysis of non-model organisms using short-read sequences. Genome Inform.

[63] Salzberg SL, Sommer DD, Puiu D, Lee VT (2008). Gene-boosted assembly of a novel bacterial genome from very short reads. PLoS Comput Biol.

[64] Toth AL, Varala K, Newman TC, Miguez FE, Hutchison SK, Willoughby DA (2007). Wasp gene expression supports an evolutionary link between maternal behavior and eusociality. Science.

[65] Bigeard J, Colcombet J, Hirt H (2015). Signaling mechanisms in pattern-triggered immunity (PTI). Mol Plant.

[66] Li B, Meng X, Shan L, He P (2016). Transcriptional regulation of pattern-triggered immunity in plants. Cell Host Microbe.

[67] Chisholm ST, Coaker G, Day B, Staskawicz BJ (2006). Host-microbe interactions: Shaping the evolution of the plant immune response. Cell.

[68] Robert-Seilaniantz A, Navarro L, Bari R, Jones JDG (2007). Pathological hormone imbalances. Curr Opin Plant Biol.

[69] Yamada S, Kano A, Tamaoki D, Miyamoto A, Shishido H, Miyoshi S (2012). Involvement of OsJAZ8 in jasmonate-induced resistance to bacterial blight in rice. Plant Cell Physiol.

[70] Berrocal-Lobo M, Molina A, Solano R (2002). Constitutive expression of Ethylene-Response-Factor1 in arabidopsis confers resistance to several necrotrophic fungi. Plant J.

[71] Van der Does D, Leon-Reyes A, Koornneef A, Van Verk MC, Rodenburg N, Pauwels L (2013). Salicylic acid suppresses jasmonic acid signaling downstream of SCFCOI1-JAZ by targeting GCC promoter motifs via transcription factor ORA59. Plant Cell.

[72] Spoel SH, Johnson JS, Dong X (2007). Regulation of tradeoffs between plant defenses against pathogens with different lifestyles. Proc Natl Acad Sci.

[73] Tamaoki D, Seo S, Yamada S, Kano A, Miyamoto A, Shishido H (2013). Jasmonic acid and salicylic acid activate a common defense system in rice. Plant Signal Behav.

[74] Mur LAJ (2005). The Outcomes of concentration-specific interactions between salicylate and jasmonate signaling include synergy, antagonism, and oxidative stress leading to cell death. Plant Physiol.

[75] Liu L, Sonbol F-M, Huot B, Gu Y, Withers J, Mwimba M (2016). Salicylic acid receptors activate jasmonic acid signalling through a non-canonical pathway to promote effector-triggered immunity. Nat Commun.

[76] Robert-Seilaniantz A, Grant M, Jones JDG (2011). Hormone crosstalk in plant disease and defense: more than just JASMONATE-SALICYLATE antagonism. Annu Rev Phytopathol.

[77] Williamson VM, Kumar A (2006). Nematode resistance in plants: the battle underground. Trends Genet.

[78] Steuernagel B, Periyannan SK, Hernández-Pinzón I, Witek K, Rouse MN, Yu G (2016). Rapid cloning of disease-resistance genes in plants using mutagenesis and sequence capture. Nat Biotechnol.

[79] Jupe F, Pritchard L, Etherington GJ, MacKenzie K, Cock PJA, Wright F (2012). Identification and localisation of the NB-LRR gene family within the potato genome. BMC Genomics.

[80] Andolfo G, Jupe F, Witek K, Etherington GJ, Ercolano MR, Jones JDG (2014). Defining the full tomato NB-LRR resistance gene repertoire using genomic and cDNA RenSeq. BMC Plant Biol.

[81] Leister D (2004). Tandem and segmental gene duplication and recombination in the evolution of plant disease resistance genes. Trends Genet.

[82] Michalak P (2008). Coexpression, coregulation, and cofunctionality of neighboring genes in eukaryotic genomes. Genomics.

[83] Yi G, Sze S-H, Thon MR (2007). Identifying clusters of functionally related genes in genomes. Bioinformatics.

[84] Rodgers-melnick E, Mane SP, Dharmawardhana P, Slavov GT, Crasta OR, Strauss SH, et al. Contrasting patterns of evolution following whole genome versus tandem duplication events in *Populus*. Genome Res. 2012:95–105. 10.1101/gr.125146.111 PMC3246211 21974993

[85] Michelmore RW, Meyers BC, Young ND (1998). Cluster of resistance genes in plants evolveby divergent selection and a birth-and-death process. Curr Opin Plant Biol.

[86] Nieri D, Di Donato A, Ercolano MR (2017). Analysis of tomato meiotic recombination profile reveals preferential chromosome positions for NB-LRR genes. Euphytica.

[87] Sinapidou E, Williams K, Nott L, Bahkt S, Tör M, Crute I (2004). Two TIR:NB:LRR genes are required to specify resistance to *Peronospora parasitica* isolate Cala2 in *Arabidopsis*. Plant J.

[88] Christie N, Tobias PA, Naidoo S, Külheim C (2016). The *Eucalyptus grandis* NBS-LRR gene family: physical clustering and expression hotspots. Front Plant Sci.

[89] Wan H, Zhao Z, Malik A, Qian C, Chen J (2010). Identification and characterization of potential NBS-encoding resistance genes and induction kinetics of a putative candidate gene associated with downy mildew resistance in *Cucumis*. BMC Plant Biol.

[90] Wan H, Yuan W, Ye Q, Wang R, Ruan M, Li Z (2012). Analysis of TIR- and non-TIR-NBS-LRR disease resistance gene analogous in pepper: characterization, genetic variation, functional divergence and expression patterns. BMC Genomics.

[91] Fuller VL, Lilley CJ, Urwin PE (2008). Nematode resistance. New Phytol.

[92] Tsuda K, Katagiri F (2010). Comparing signaling mechanisms engaged in pattern-triggered and effector-triggered immunity. Curr Opin Plant Biol.

[93] Yang S, Li J, Zhang X, Zhang Q, Huang J, Chen J (2013). Rapidly evolving R genes in diverse grass species confer resistance to rice blast disease. Proc Natl Acad Sci.

