# Supplementary figures and images for: Comparative root transcriptome of wild Arachis reveals NBS-LRR genes related to nematode resistance
Source: BMC Plant Biol. 2018 Aug 6;18:159. doi: 10.1186/s12870-018-1373-7 (PMC6080386; doi:10.1186/s12870-018-1373-7)

Color Key  
and Histogram

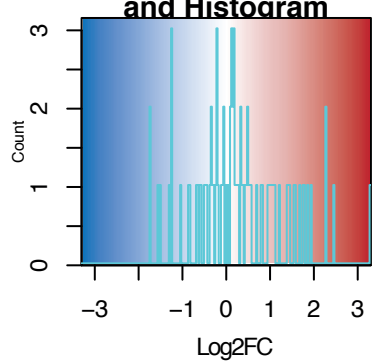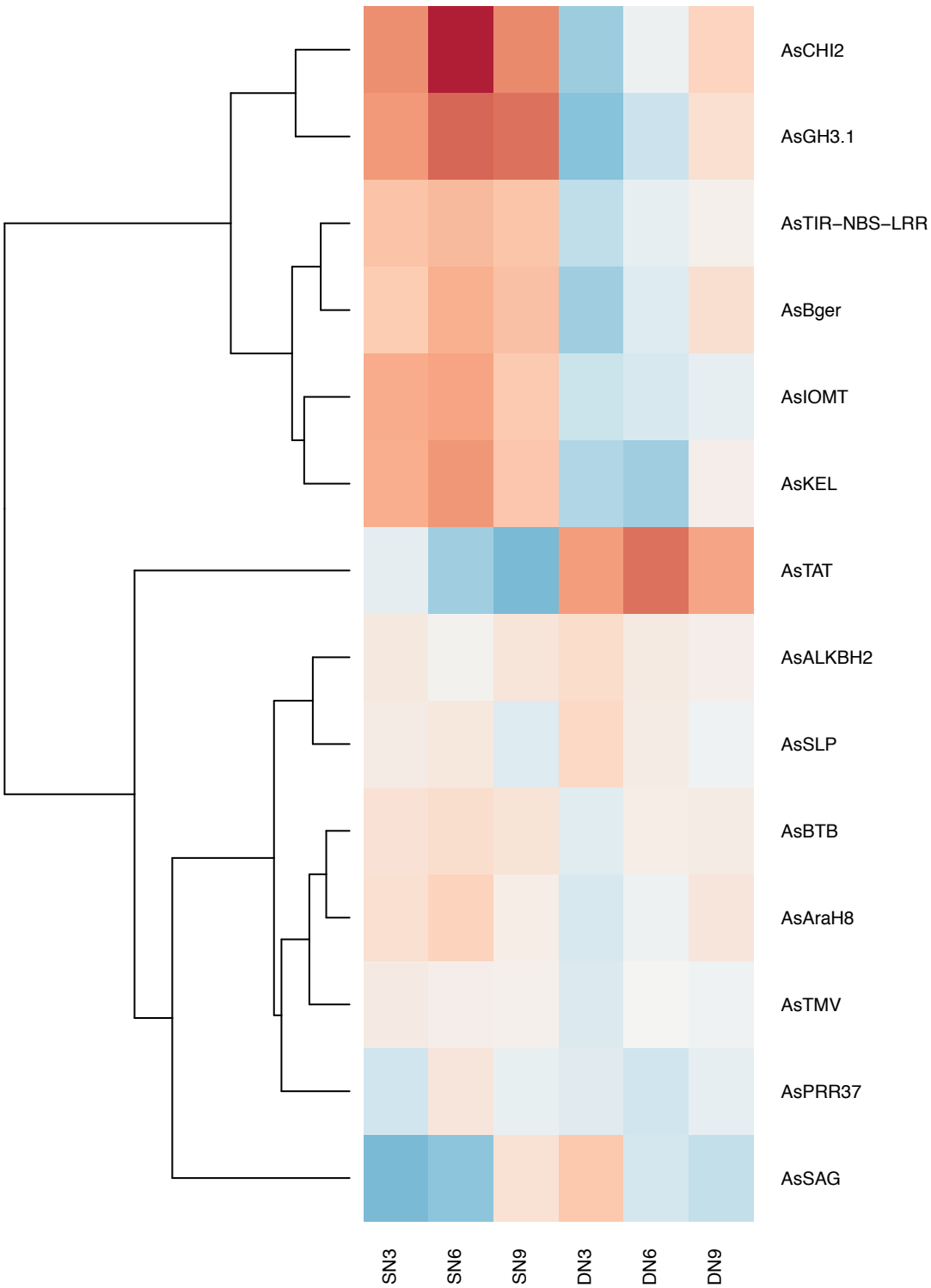

Supplement: Supplementary file 3 — Figure S1. Expression profiles of 14 nematode responsive candidate genes in A. stenosperma and A. duranensis at 3,6 and 9 DAI with M. arenaria. (PDF 46 kb) [file 12870_2018_1373_MOESM3_ESM.pdf]

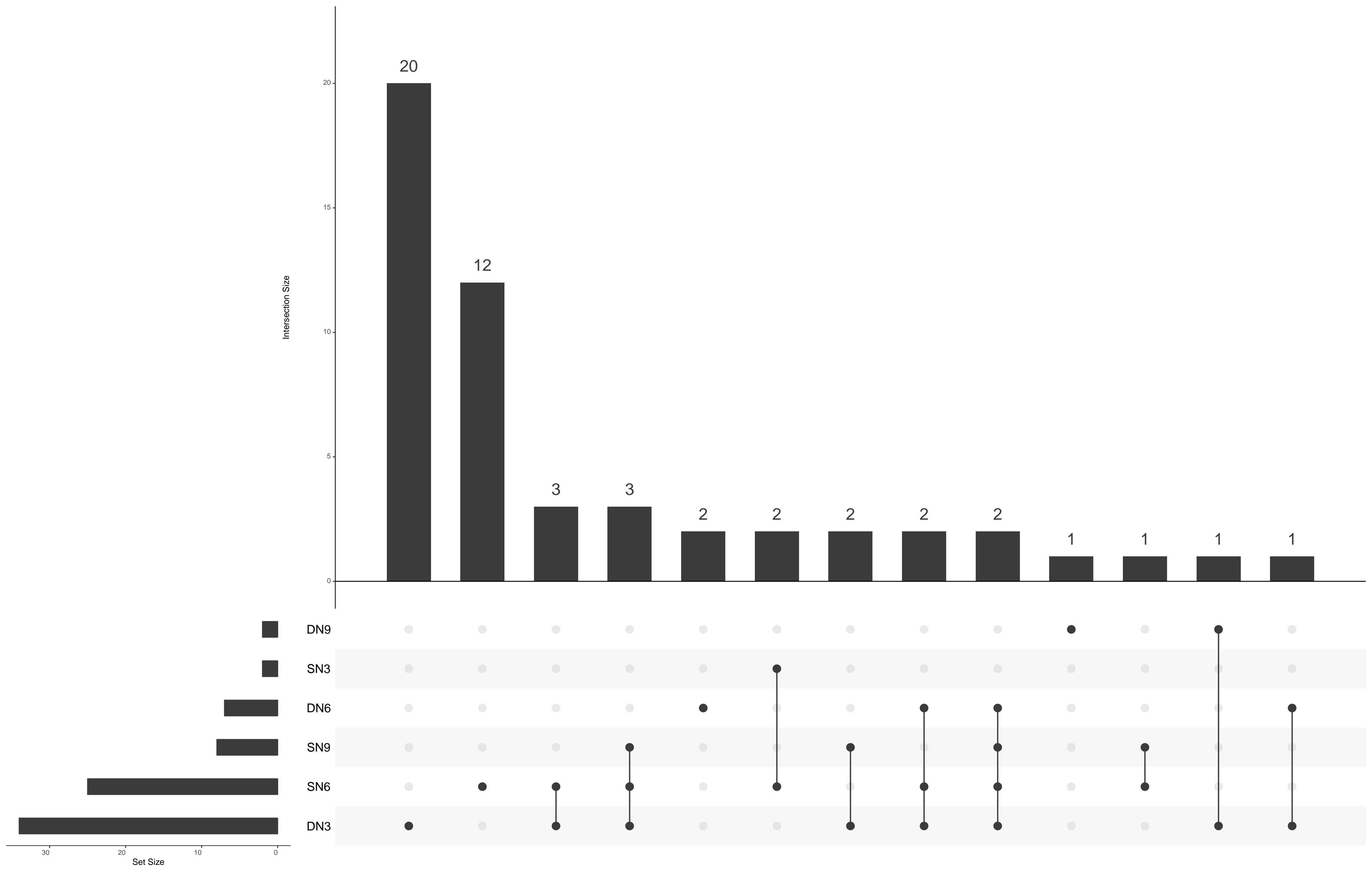

Supplement: Supplementary file 5 — Figure S3. Intersections between the NBS-LRR (LDEG) (FDR<0.05) in A. duranensis (DN3, DN6, DN9) and A. stenosperma (SN3, SN6, SN9) infected with M. arenaria. The set size represents the number of RKN- responsive genes in each condition (genotype/DAI) and the black dots their intersections. (PDF 8 kb) [file 12870_2018_1373_MOESM5_ESM.pdf]

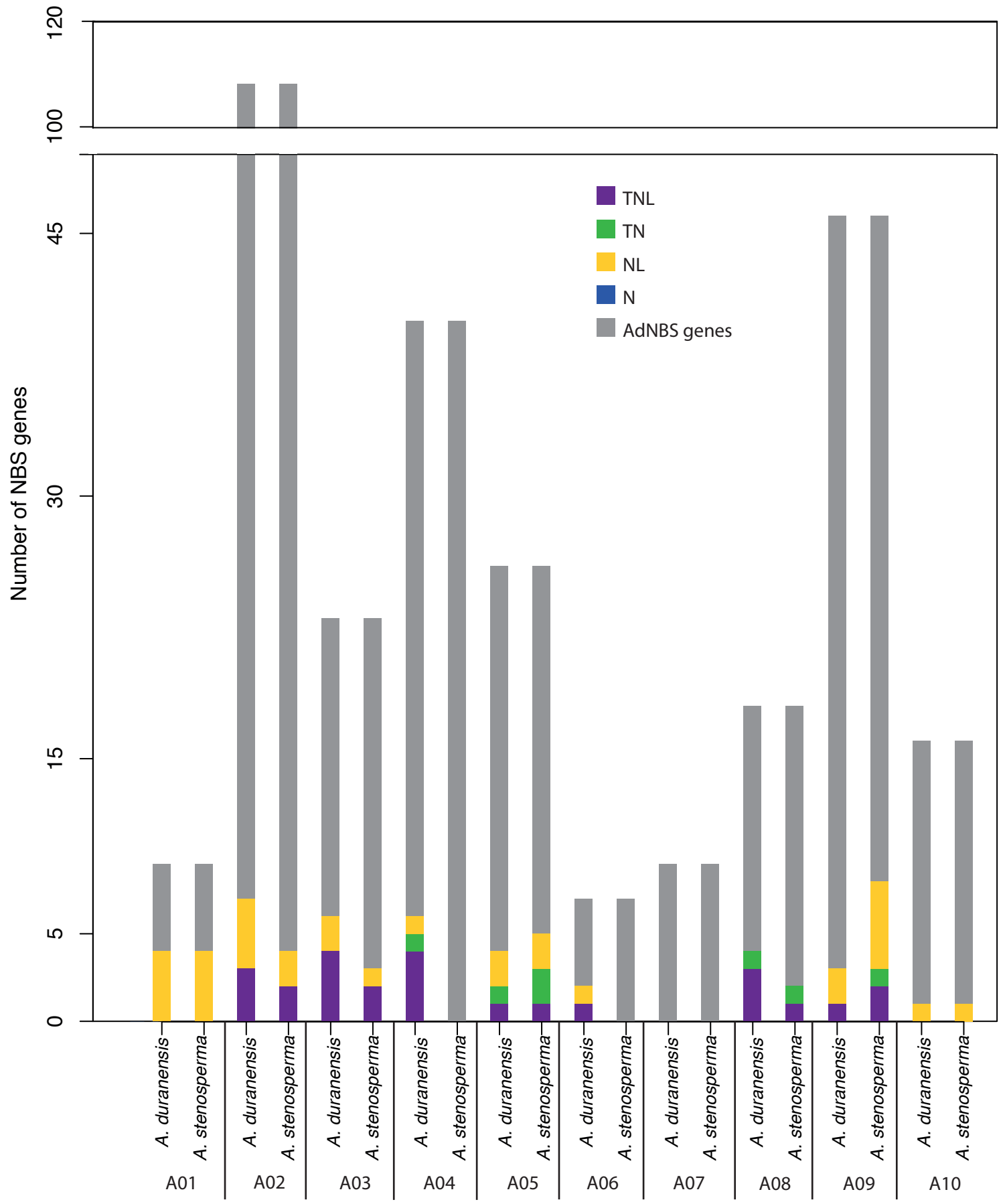

Supplement: Supplementary file 7 — Figure S4. Distribution of A. stenosperma and A. duranensis expressed NBS-LRR subclasses in A. duranensis chromosomes (http://peanutbase.org/). (PDF 1178 kb) [file 12870_2018_1373_MOESM7_ESM.pdf]
