# Supplementary material for: Comparative root transcriptome of wild Arachis reveals NBS-LRR genes related to nematode resistance
Source: BMC Plant Biol. 2018 Aug 6;18:159. doi: 10.1186/s12870-018-1373-7 (PMC6080386; doi:10.1186/s12870-018-1373-7)
Supplement: Supplementary file 4 — Figure S2. Transcriptional profile of members of four TF families: (A) WRKY (B) MYB (C) bZIP (D) ERF in A. stenosperma (SN3, SN6, SN9) and A. duranensis (DN3, DN6, DN9) in response to RKN infection. (PDF 1356 kb) [file 12870_2018_1373_MOESM4_ESM.pdf]

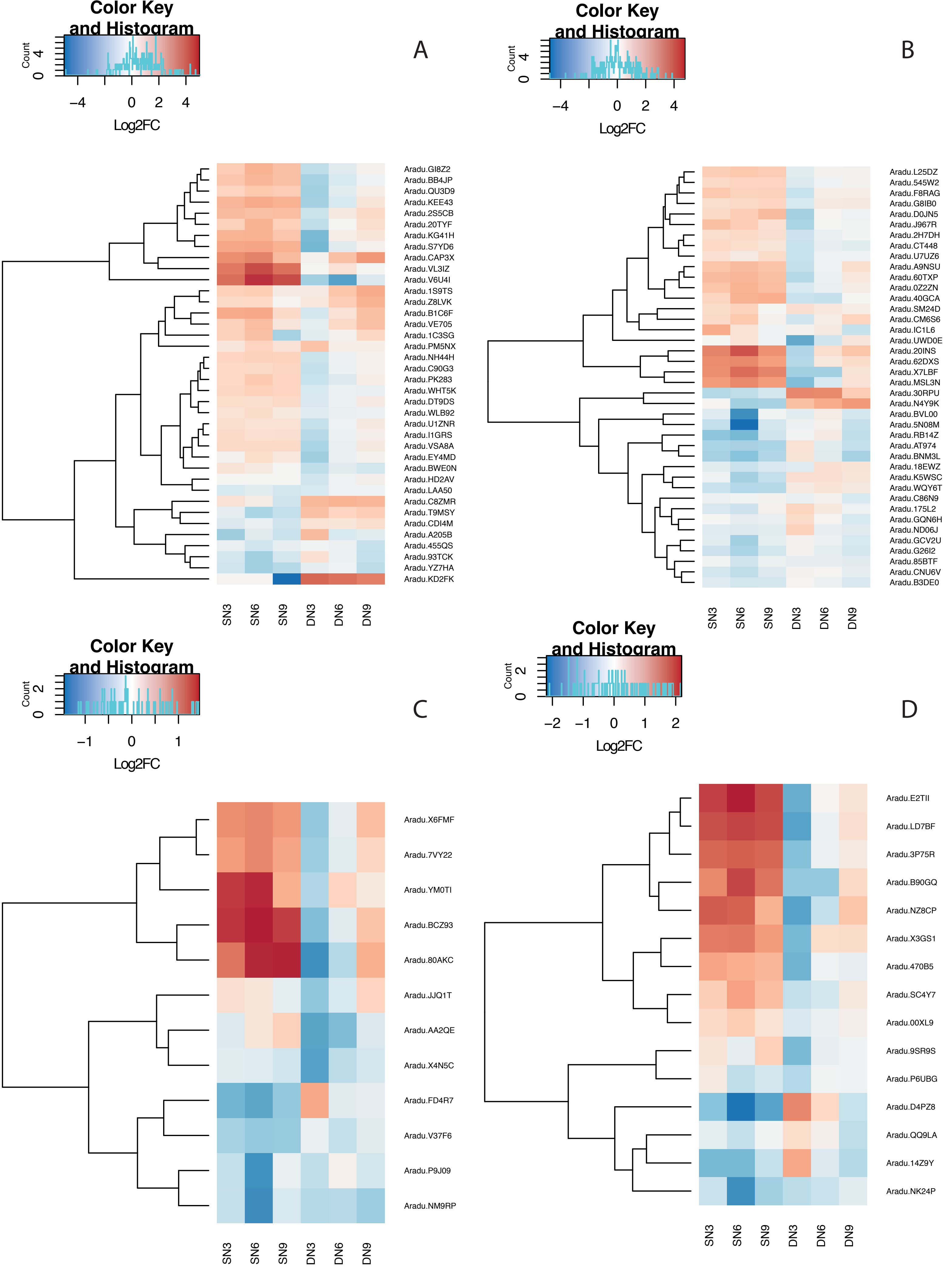
A
Color Key and Histogram
Count
0
4
−4
0
2
4
Log2FC
Aradu.GI8Z2
Aradu.BB4JP
Aradu.QU3D9
Aradu.KEE43
Aradu.2S5CB
Aradu.20TYF
Aradu.KG41H
Aradu.S7YD6
Aradu.CAP3X
Aradu.VL3IZ
Aradu.V6U4I
Aradu.1S9TS
Aradu.Z8LVK
Aradu.B1C6F
Aradu.VE705
Aradu.1C3SG
Aradu.PM5NX
Aradu.NH44H
Aradu.C90G3
Aradu.PK283
Aradu.WHT5K
Aradu.DT9DS
Aradu.WLB92
Aradu.U1ZNR
Aradu.I1GRS
Aradu.VSA8A
Aradu.EY4MD
Aradu.BWE0N
Aradu.HD2AV
Aradu.LAA50
Aradu.C8ZMR
Aradu.T9MSY
Aradu.CDI4M
Aradu.A205B
Aradu.455QS
Aradu.93TCK
Aradu.YZ7HA
Aradu.KD2FK
SN3
SN6
SN9
DN3
DN6
DN9
B
Color Key and Histogram
Count
0
4
−4
0
2
4
Log2FC
Aradu.L25DZ
Aradu.545W2
Aradu.F8RAG
Aradu.G8IB0
Aradu.D0JN5
Aradu.J967R
Aradu.2H7DH
Aradu.CT448
Aradu.U7UZ6
Aradu.A9NSU
Aradu.60TXP
Aradu.0Z2ZN
Aradu.40GCA
Aradu.SM24D
Aradu.CM6S6
Aradu.IC1L6
Aradu.UWD0E
Aradu.20INS
Aradu.62DXS
Aradu.X7LBF
Aradu.MSL3N
Aradu.30RPU
Aradu.N4Y9K
Aradu.BVL00
Aradu.5N08M
Aradu.RB14Z
Aradu.AT974
Aradu.BNM3L
Aradu.18EWZ
Aradu.K5WSC
Aradu.WQY6T
Aradu.C86N9
Aradu.175L2
Aradu.GQN6H
Aradu.ND06J
Aradu.GCV2U
Aradu.G26I2
Aradu.85BTF
Aradu.CNU6V
Aradu.B3DE0
SN3
SN6
SN9
DN3
DN6
DN9
C
Color Key and Histogram
Count
0
2
−1
0
1
Log2FC
Aradu.X6FMF
Aradu.7VY22
Aradu.YM0TI
Aradu.BCZ93
Aradu.80AKC
Aradu.JJQ1T
Aradu.AA2QE
Aradu.X4N5C
Aradu.FD4R7
Aradu.V37F6
Aradu.P9J09
Aradu.NM9RP
SN3
SN6
SN9
DN3
DN6
DN9
D
Color Key and Histogram
Count
0
2
−2
−1
0
1
2
Log2FC
Aradu.E2TII
Aradu.LD7BF
Aradu.3P75R
Aradu.B90GQ
Aradu.NZ8CP
Aradu.X3GS1
Aradu.470B5
Aradu.SC4Y7
Aradu.00XL9
Aradu.9SR9S
Aradu.P6UBG
Aradu.D4PZ8
Aradu.QQ9LA
Aradu.14Z9Y
Aradu.NK24P
SN3
SN6
SN9
DN3
DN6
DN9
